# The Baltimore College of Dental Surgery—Female Students

**Published:** 1873-01

**Authors:** 


### EDITORIAL, ETC.

*The Baltimore College of Dental Surgery—Female Students.*—The friends of this pioneer institution in dental education will be pleased to learn that the present session is the most successful one since the late war—the class now attending lectures being larger than during any session since 1861. This we take for a happy augury that the financial affairs of the Southern States are improving, as the great majority of the students attending this College are Southerners; nearly every Southern State being represented, as well as several Northern ones, and also a number of European countries.

Among the matriculants are two ladies from the North German Empire, one of whom is now attending her second course, and will be a candidate for graduation in March next.

The experiment of receiving female students, inaugurated last session, has proven so successful, and the effect upon the male students so beneficial, that the Faculty are perfectly satisfied with their action in this respect.

The greatest respect and consideration has, at all times, been shown by the male members of the class to these lady students, their behaviour being such as to reflect honor upon themselves, and to merit the title of gentlemen.

